# The Evolution of Neo-Adjuvant Therapy in the Treatment of Oesophageal and Gastro-Oesophageal Junction Adenocarcinomas

**DOI:** 10.3390/cancers15194741

**Published:** 2023-09-27

**Authors:** Alexander A. Dermanis, Sivesh K. Kamarajah, Benjamin Tan

**Affiliations:** 1 Queen Elizabeth Hospital, Mindelsohn Way, Birmingham B15 2GW, UK; alexdermanis@yahoo.co.uk (A.A.D.);; 2 Academic Department of Surgery, Institute of Applied Health Research, University of Birmingham, Birmingham B15 2TT, UK

**Keywords:** neoadjuvant, chemotherapy, radiotherapy, immunotherapy, adenocarcinoma, oesophagus, gastro-oesophageal junction

## Abstract

**Simple Summary:**

This is a review that provides an overview of neoadjuvant therapy, landmark clinical trials, and further prospective management options which can be considered for adenocarcinoma of the oesophagus and gastro-oesophageal junction.

**Abstract:**

Historically, oesophageal and gastro-oesophageal junction adenocarcinomas were associated with a poor prognosis. The advent of neoadjuvant therapy has transformed the management of oesophageal and gastro-oesophageal junction adenocarcinomas further and offers the possibility to reverse disease progression, eliminate micrometastasis, and offer potentially better outcomes for these patients. This review provides an overview of landmark clinical trials in this area, with different treatment regimens considered over the years as well as potential therapeutic agents on the horizon that may transform the management of oesophageal and gastro-oesophageal junction adenocarcinomas further.

## 1. Introduction

Traditionally oesophageal and gastro-oesophageal junction adenocarcinomas were associated with poor prognosis due to their insidious onset and advanced nature at the time of diagnosis [1]. The advent of neoadjuvant therapy has transformed the management of oesophageal and gastro-oesophageal junction adenocarcinomas. In cases of locally advanced and borderline resectable adenocarcinoma which may have previously been considered inoperable, neoadjuvant therapy offers the possibility to downstage disease progression, as well as reverse micrometastasis, offering potentially better outcomes for these patients. This is reflected in the literature with commonly cited figures demonstrating a gradual improvement in 5-year survival rates for these patients from 12–19% in the 1970s [2,3,4] to 39–47% today [2,3,5]. Whilst this improvement in outcomes can be accounted for by better patient selection and advances in surgical technique, the advent of neoadjuvant therapy as a standard of care is likely to be a significant contributing factor. In recent years, with the introduction of immunotherapy into treatment regimens, there is potential to improve clinical outcomes further with meticulous patient selection and multi-disciplinary input. This review provides an overview of landmark clinical trials in this area, with different treatment regimens considered over the years as well as potential therapeutic agents on the horizon that may transform the management of oesophageal and gastro-oesophageal junction adenocarcinomas further.

## 2. Neoadjuvant Chemotherapy

The origins of neoadjuvant chemotherapy trace back to the 1980s and early 1990s, when multiple small clinical trials were underway to investigate the potential therapeutic benefits of neoadjuvant therapy. The majority of these trials were largely based on cisplatin or Carboplatin regimens often using a 5-fluorauracil infusion as dual or triple therapy [6,7]. Indeed, the results of many of these trials initially demonstrated no significant difference in long-term survival with neoadjuvant therapy. In particular, the North American INT 113 trial performed from 1990 to 1995 initially demonstrated no significant difference in survival outcomes between 440 patients with stage I, II, and III oesophageal cancer who were randomised to three cycles of cisplatin and 5-fluorouracil with surgery and surgery alone [8]. However, a long-term follow-up of patients in this cohort did demonstrate encouraging survival results for patients with disease clear margins (R0) post-operatively [9]. This, alongside the statistically significant reduced R1 resection rate found in patients randomised to the neoadjuvant chemotherapy wing [8,9] continued to pique interest in the topic.

Concurrently, other landmark clinical trials soon came to dominate the academic landscape in this area. Namely, the Medical Research Council Oesophageal Cancer Working Group published their research paper in 2002 [10]. This study, OEO2, which took place between 1992 and 1998, included 802 patients with oesophageal cancer, of whom were randomised to two 4-day cycles of cisplatin (80 mg/m^2^) with fluorouracil (1000 mg/m^2^) as a neoadjuvant regimen prior to surgical intervention versus surgical resection alone. Median follow-up for the neo-adjuvant group was 36.9 months versus 37.9 months for the comparator. Their findings identified an improved R0 rate in the neoadjuvant chemotherapy group (60% vs. 54%; *p* < 0.0001), as well as improved 2-year survival (43% vs. 34%; *p* < 0.05) [10]. Whilst only 70% of their patients had adenocarcinoma, their subgroup analysis demonstrated no evidence of change in response to chemotherapy with differing histology [10]. Long-term follow-up demonstrated a statistically significant sustained improvement in survival, with 5-year survival in the chemotherapy group reported at 23.0% compared to 17.1% [11]. This landmark trial was one of the first of its size to demonstrate a statistically significant survival benefit from the use of neo-adjuvant chemotherapy in the oesophageal cancer population.

The MAGIC trial also demonstrated encouraging results. This trial, published in 2006, randomised 503 patients with lower oesophageal, gastric-oesophageal junction (GOJ), and gastric adenocarcinoma to a neoadjuvant regimen of 3, three-weekly cycles, consisting of epirubicin (50 mg/m^2^) and cisplatin (60 mg/m^2^) on day one, followed by a fluorouracil infusion (200 mg/m^2^) for 21 days, compared with surgery alone [12]. With a median follow-up of 4 years, the investigators found a statistically significant increase in overall survival for the chemotherapy group (hazard ratio [HR] 0.75; 95% CI [confidence interval] 0.60 to 0.9; *p* = 0.009), as well as a higher reported five-year survival rate (36% vs. 23%) [12]. The limitations of this trial include the inclusion of gastric adenocarcinoma in the patient cohort, which formed 74% of the included patients, making generalising conclusions about oesophageal and GOJ adenocarcinoma difficult. The 2011 French FNCLCC/FFCD 9703.3 study, conducted between 1995 to 2003, randomised 224 patients from 28 centers to a similar chemotherapy regimen consisting of two or three preoperative 4-week cycles of intravenous cisplatin (100 mg/m^2^) on day 1, and a continuous intravenous infusion of fluorouracil (800 mg/m^2^/d) for days 1–5. This was followed by three or four postoperative cycles of the same regimen as in the comparator following surgery [13]. As with MAGIC, they found overall survival was improved in the neoadjuvant chemotherapy group (HR 0.69; 95% CI 0.50 to 0.95; *p* = 0.02), with a 5-year survival rate of 38% vs. 24%, respectively. They additionally found improved statistically significant disease-free survival at 5 years (34% vs. 19%; HR, 0.65; 95% CI 0.48 to 0.89; *p* = 0.003). Similar to MAGIC, the inclusion of gastric adenocarcinoma patients in 74% and 25% of the intervention and comparator wings, respectively, affects the generalisability of these findings.

Cisplatin chemotherapy is associated with notable side effects, namely, nephrotoxicity, peripheral neuropathy, hyperemesis, and myelosuppression. This led to a drive to incorporate newer agents into neoadjuvant regimens, which were thought to be more efficacious and hypothetically have better side effect profiles. The docetaxel, cisplatin, and 5-Fluorouracil (DCF) regimen has been trialled in some studies and is thought to demonstrate some survival benefits; however, largely, these have been small studies limited to malignancies with squamous cell carcinoma [14,15]. In 2011, a phase II trial conducted by Ferri et al. investigated 43 patients with adenocarcinoma of the oesophagus who received 3 cycles of pre- and postoperative DCF chemotherapy consisting of 75 mg/m^2^ of docetaxel, cisplatin on day 1 and 750 mg/m^2^/day of 5-fluorouracil on continuous perfusion from days 1 to 5 [16]. Their findings identified that the DCF regimen was a tolerable chemotherapy regimen with acceptable long-term outcomes, identifying a 3-year survival rate of 60% of all patients. A retrospective study by Fiteni et al. in 2016 compared 62 patients who received at least one cycle of a perioperative DCF regimen (identical to Ferri et al.) to 789 patients who had surgery alone, every 3 weeks [17]. In contrast with the surgery group, the DCF group demonstrated a statistically significant overall survival benefit, with a HR of 0.29 (95% IC, 0.14–0.64) in the matched-pair analysis and 0.59 (95% CI, 0.45–0.78) in the IPTW analysis. However, whilst an improvement in the R0 resection rate was noted, this was not statistically significant. The small number of patients in the intervention arm, the retrospective nature of the study, and the lack of data on toxicity in the study limit any meaningful conclusions.

More recently, the FLOT-4 trial, published in 2019, investigated a new regimen of 4 pre- and postoperative 2-week cycles of fluorouracil (2600 mg/m^2^), leucovorin (100 mg/m^2^), oxaliplatin (85 mg/m^2^) and docetaxel (50 mg/m^2^) as a continuous regimen (FLOT). This was compared with 6 perioperative 3-week cycles of epirubicin (50 mg/m^2^) and cisplatin (60 mg/m^2^) on day 1 and either fluorouracil (200 mg/m^2^) as a continuous infusion or oral capecitabine (1250 mg/m^2^) on days 1 to 21 (ECF/ECX), as based on the MAGIC regimen [18]. This study recruited 716 patients across 36 centres during the years 2010 to 2015 and found that overall survival was increased in the FLOT group compared with the ECF/ECX group (HR 0.77; 95% CI 0.63 to 0.94; *p* = 0.012). Their analysis indicated that for the FLOT group median, overall survival increased by 50 months (38.33 to not reached), whereas for the ECF/ECX group median overall survival increased by 35 months (27.35 to 46.26). A higher proportion of patients achieved R0 outcomes in the FLOT group than in the ECF/ECX group (301 patients [85%] vs. 279 patients [78%]; *p* = 0.0162). The estimated overall 5-year survival for the FLOT group was 45% (38 to 51) and for the ECF/ECX group was 36% (30 to 42). As expected, given the different chemotherapy regimens the overall side effect picture was different, with the most frequent grade 3 or 4 complications being neutropenia, observed in approximately 40% of patients treated with ECF/ECX versus 50% of patients treated with FLOT. FLOT treatment caused markedly less grade 3 and 4 nausea (7% vs. 16%), most likely due to the use of oxaliplatin instead of emetogenic cisplatin. On the other hand, FLOT caused more infections (18% vs. 9%) and diarrhoea (10% vs. 4%). There was no variation in toxic deaths, admission for chemotherapy-related side effects, or discontinuation when comparing both regimens. Overall, these findings indicated a possible survival benefit for gastro-oeosphageal junction-related tumours with the FLOT regimen.

As more evidence emerged demonstrating the benefit of neoadjuvant chemotherapy, meta-analyses were also underway to evaluate the use of neoadjuvant chemotherapy in this group. In 2008, a meta-analysis by Gebski et al. found a statistically significant survival benefit comparing neoadjuvant chemotherapy to surgery alone (HR 0.90; 95% CI 0.81 to 1.00; *p* = 0.05), with a 2-year absolute survival benefit of 7% and number needed to treat of as 15 [19]. This meta-analysis was updated in 2011 and demonstrated a continued statistically significant overall survival benefit (HR 0.87; 95% CI 0.79 to 0.96; *p* = 0.005), with a 2-year absolute survival difference of 5.1% and a number needed to treat of 19 [20]. More recently, a network meta-analysis in 2018 by Chan et al. showed a trend toward survival benefits for neoadjuvant chemotherapy, but this did not reach statistical significance [21]. This was confirmed by the 2020 systematic review by Kumar et al. who also did not find a statistically significant survival benefit with neoadjuvant chemotherapy [22]. Unlike previous analyses, Kumar et al. excluded any perioperative regimen and therefore utilised only truly neoadjuvant therapy. They did, however, find a statistically significant survival benefit with neoadjuvant chemoradiotherapy which sets the scene for the possible adoption of chemoradiotherapy as the mainstay of neo-adjuvant treatment.

## 3. Neoadjuvant Chemoradiotherapy

Currently, neoadjuvant chemoradiotherapy in most Western countries is the gold standard regimen in managing oesophageal and gastro-oesophageal junction malignancies [23]. The first reasonably sized trial to demonstrate the benefit of chemoradiotherapy in this group was the RTOG 8501 trial, which occurred between 1985 and 1990, randomised 129 patients between chemoradiotherapy and radiotherapy alone. The trial was stopped after results demonstrated that the chemoradiotherapy wing, containing a regimen of cisplatin and a continuous infusion of fluorouracil, demonstrated a survival benefit, as well as fewer local and systemic recurrences [24]. However, as expected, this trial also demonstrated more severe side effects, occurring in 19% of patients in comparison with radiotherapy alone [24]. At this time, the superiority of chemoradiotherapy to other treatment regimens was corroborated by Walsh et al., who published their findings in 1996 [25]. They compared a neoadjuvant chemoradiotherapy regimen consisting of two cycles of 5-fluorouracil (15 mg/kg) infused on day 1 through 5, cisplatin (75 mg/m^2^) on day 7 and 15 fractions of radiotherapy (40 Gray [Gy]) with surgery alone for managing oesophageal adenocarcinoma [25]. With 113 patients randomised to either intervention wing, they discovered a statistically significant 3-year survival benefit, with 32% of patients alive in the chemoradiotherapy wing versus 6% of patients alive in the surgical wing (*p* = 0.01) [25].

The 2008 Dutch CROSS trial was the first large-scale RCT to compare neoadjuvant chemoradiotherapy with surgery alone [26]. This study randomised 368 patients to surgery alone or surgery with a neoadjuvant chemoradiotherapy regimen consisting of five weekly cycles of carboplatin (titrated to achieve an area under the curve of 2 mg per ml per minute) and paclitaxel (50 mg/m^2^), alongside 41.4 Gy in 23 fractions of radiotherapy, 5 days per week. Median survival was greater in the chemoradiotherapy group, at 49.4 months versus 29.4. There was a statistically significant improvement in overall survival, which was substantiated with long-term follow-up (HR 0.657; 95%CI: 0.495 to 0.871; *p* = 0.003) [26]. Pathological complete response (pCR) was achieved in 29% of patients in the chemoradiotherapy wing. Of note, one patient died while following chemoradiotherapy, due to a major haemorrhage from an aorto-oesophageal fistula in the absence of thrombocytopenia. However, the most frequent grade 3 or higher adverse effect was leukoplakia, reported in 6% of patients. Long-term follow-up demonstrated 3-year and 5-year survival rates at 58% (51–65) and 47% (39–54) for the neoadjuvant arm versus 44% (37–51), and 33% (26–40) for the surgery alone arm [27]. These findings were observed in adenocarcinoma, which formed 75% of the cohort. The epochal findings of the CROSS trial led to neoadjuvant chemoradiotherapy becoming adopted as a gold standard.

Despite this, there remains a paucity of literature in this area for studies evaluating the effect of chemoradiotherapy in oesophageal adenocarcinoma alone. The NEOCRTEC5010 trial published in 2018 only evaluated neoadjuvant chemoradiotherapy in squamous cell carcinoma [28]. The 2014 FFCD 9901 trial evaluated a neoadjuvant chemoradiotherapy regimen consisting of 2 cycles of 5-fluorouracil (continuous infusion from days 1 to 4, 29 to 32; 800 mg/m^2^ per 24 h), alongside 4 days of cisplatin (75 mg/m^2^) and 45 Gy of radiotherapy delivered in 25 fractions over 5 weeks for stage I or II oesophageal cancer [29]. This study was also limited by its small numbers of adenocarcinoma, accounting for only 29% of the 195 patients studied. Its findings demonstrated an R0 rate of 93.8% versus 92.1% (*p* = 0.749), a 3-year survival rate of 47.5% versus 53.0% (HR 0.99; 95% CI 0.69 to 1.40; *p* = 0.94) and postoperative mortality rate of 11.1% versus 3.4% (*p* = 0.049), respectively. Its futility could be explained by the large numbers of squamous cell carcinoma, which have demonstrated mixed responses to chemoradiotherapy in the CROSS trial [27], but also the differences in chemotherapy regimens, patient selection, and more tolerable lower doses of radiotherapy used in the CROSS trial may further underly these differences.

Another potential neoadjuvant option that is being considered for patients with locally advanced disease is a regimen consisting of induction chemotherapy, chemoradiotherapy, and then surgical resection. The addition of induction chemotherapy is hypothesised to have a further therapeutic effect on malignancies that are chemoradiotherapy responsive, causing additional disease regression and hypothetically better surgical outcomes at the expense of potentially more frequent and severe side effects. The NCCTG N0849 trial, published in 2021 compared a triple regimen, consisting of an induction regimen of docetaxel (60 mg/m^2^, day 1), oxaliplatin (85 mg/m^2^, day 1), and capecitabine (625 mg/m^2^, day 1–14) every 21 days for two cycles followed by a neoadjuvant regimen consisting of a 5-day 5-fluorouracil infusion (180 mg/m^2^/day), oxaliplatin (85 mg/m^2^) and daily radiotherapy (50.4 Gy in 28 fractions) and then surgical resection, with identical neoadjuvant chemoradiotherapy and surgery alone [30]. In their study, the pCR rate was 28.6% in the intervention wing versus 40.7% in the comparator (*p* = 0.34). During induction chemotherapy, 57.1% of patients experienced neutropenia. The rates of grade 3–5 toxicity in chemoradiation in the intervention wing and comparator were 32.2% and 66.7%, respectively. Three deaths were to be related to treatment: one colitis, one cardiac arrest during induction chemotherapy, and one postoperative sepsis in the comparator. Given no statistical differences were observed in outcomes, the trial was terminated, but patients were followed up. After a median follow-up of 60.4 months, a longer median OS was unexpectedly observed, with 3-year rates at 57.1% versus 41.7%, respectively. This was driven by longer DFS after margin-free surgery, among patients with well/moderately differentiated tumours, but not among patients with poorly/undifferentiated tumours. One possible explanation for these findings could be due to the accumulation of chemotoxicity, resulting in reduced fluorouracil uptake in the intervention arm—81% (71–96%) versus 86% (77–100%). However, other studies in this area have also demonstrated no statistically significant change in pathological complete response or overall survival, despite reporting minimal toxicity [31]. Further evaluation of the intensification of pre-operative chemotherapy is needed to evaluate who, if any patients may benefit from this intervention and how this regimen is best delivered.

## 4. Neoadjuvant Chemoradiotherapy vs. Neoadjuvant Chemotherapy

The choice between neoadjuvant chemotherapy versus chemoradiotherapy remains contentious. Several studies have sought to address this issue and identify an overall consensus. The POET trial, which took place from November 2000 until December 2005, included 126 patients from 19 German centres [32,33]. The neoadjuvant chemotherapy (nCT) wing consisted of 2.5 cycles of a 6-week schedule of a 7-day fluorouracil infusion (2 g/m^2^, 24 h), leucovorin infusion (500 mg/m^2^, 2 h) as well as biweekly cisplatin (50 mg/m^2^). In the neoadjuvant chemoradiotherapy (nCRT) wing, the same 2 cycles of this regimen were followed 2 weeks later by a course of cisplatin (50 mg/m^2^) on days 1 and 8 and etoposide (80 mg/m^2^) on days 3 to 5. A total dose of 30 Gy of radiotherapy was given at 2.0 Gy per fraction over 15 weeks. The chemoradiotherapy group showed a significantly higher pCR rate (15.6% vs. 2.0%, *p* = 0.03) and tumour-free lymph nodes (64.4% vs. 27.7%, *p* = 0.01). In spite of the fact that the nCRT arm showed a large but statistically insignificant trend toward a 3-year survival benefit, the long-term results still suggested a superiority in local progression-free survival (PFS) in the nCT arm [33].

Consistent with the observations in the POET trial, higher pCR and R0/R1 was also favoured by the nCRT arm in both the multicentre Neo-Res and Australian trials [34,35,36]. However, all of these trials failed to show a long-term statistical survival benefit with neoadjuvant chemoradiotherapy [34,35,36].

In order to determine the best neoadjuvant regimen, a network meta-analysis including 26 studies was completed compared the efficacy of surgery alone with nCT, neoadjuvant radiotherapy, nCRT, surgery followed by adjuvant chemotherapy, adjuvant radiotherapy, or adjuvant chemoradiotherapy. In their ranking analysis, they identified that nCRT might be the best option for patients diagnosed with locally advanced oesophageal cancer. When compared to surgery alone, in all the treatments, nCRT yielded the greatest benefit in terms of OS and PFS/DFS (HR = 0.76, 95% CI 0.67 to 0.85; HR = 0.8, 95% CI 0.68 to 0.94, respectively). Only nCRT was associated with a statistically confident decrease in locoregional recurrence or distant metastasis (Odds Ratio [OR] 0.48, 95% CI 0.30–0.77; OR = 0.67, 95% CI 0.49–0.93, respectively) [37].

Therefore, whilst there is some emerging evidence that neoadjuvant chemoradiotherapy may provide a greater benefit compared with other neoadjuvant regimens for locally advanced oesophageal cancer, there is still no overall consensus on the optimal regimen for oesophageal adenocarcinoma just yet. Furthermore, the addition of radiotherapy needs to be balanced with the risk of toxicity side effects, such as radiation pneumonitis and cardiac toxicity. The CROSS trial did use a lower dose of radiotherapy (41.4 Gy in 23 fractions) [26] and there remains a paucity of literature on the optimal radiation dose for this regimen. Furthermore, none of the previously mentioned studies directly compared chemotherapy regimens according to MAGIC, OEO2, or FLOT trials with nCRT according to CROSS, despite these being the most widely used regimens in clinical practice. A number of current ongoing trials are therefore continuing to evaluate the use of chemoradiotherapy to establish the optimum regimen. The Neo-AEGIS trial is due to be published, which has compared nCRT according to the CROSS regimen with a modified perioperative MAGIC regimen [38]. The phase III randomised ESOPEC trial is ongoing currently and will compare perioperative FLOT with CROSS-based nCRT [39]. Finally, another important consideration is in patients where pCR is achieved by neoadjuvant therapy alone, as it may be appropriate to consider active surveillance rather than surgical resection. The Netherlands SANO trial, France ESOSTRATE and Chinese CELAEC are ongoing RCTs assessing active surveillance in this pCR cohort [40,41].

## 5. Immunotherapy

Interest in the use of monoclonal antibodies has grown in recent years. Biomarkers, such as Epidermal Growth Factor Receptor, Erb-B2 Receptor Tyrosine Kinase 2 (HER2), and Vascular Endothelial Growth Factor Receptor, have all been reported in oesophageal cancer [42].

**Figure 1:**
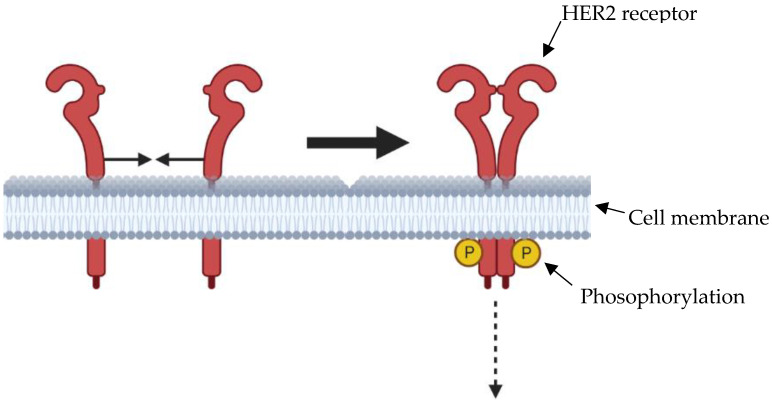
Overexpression of HER2 receptors on the cell surface, leads to receptors dimerising more frequently, causing autophosphorylation and activation of intracellular signalling processes promoting cell cycle progression and therefore carcinogenesis. This is therefore a potential target for monoclonal antibodies.

Trastuzumab is a monoclonal antibody that has a mechanism of action where it binds to and inhibits the Human Epidermal growth factor Receptor 2 (HER2/neu), which is responsible for the proliferation and inhibition of apoptosis of the cell (Figure 1). In patients with metastatic breast cancer, the use of trastuzumab resulted in good responses with only mild to moderate side effects [43]. Interest in trastuzumab in managing oesophagogastric cancer was largely a result of the study by Bang et al. [44]. This study randomly allocated 594 patients with advanced gastric or gastroesophageal junctional cancer to an intervention wing consisting of trastuzumab plus chemotherapy versus a comparator, namely chemotherapy alone. The median follow-up was comparable, namely 18.6 months for patients undergoing trastuzumab and chemotherapy versus 17.1 months for patients undergoing chemotherapy alone. Median OS significantly improved from 11.1 months to 13.8 months with the addition of Trastuzumab (HR 0.74; 95% CI, 0.60–0.91, *p* = 0.0046). Rates of adverse events (201 [68%] vs. 198 [68%]) did not differ between groups. Finally, combination therapy with two immunological agents may be considered. Further studies, such as the KEYNOTE118 and the TRAP trial are ongoing to evaluate a combination of trastuzumab and another immunological agent in gastric cancer, the latter of which has shown promising results in an initial phase II trial for HER2-positive patients [45,46].

Cetuximab is another monoclonal antibody, which binds and inhibits the Epidermal Growth Factor Receptor (EGFR) which has similar properties to HER2/neu. The Swiss Group for Clinical Cancer Research (SAKK) has performed trials using cetuximab in oesophageal cancer treatment. Firstly, the phase Ib/II-SAKK 75/06 trial indicated that the addition of cetuximab to induction chemotherapy followed by neoadjuvant chemoradiotherapy demonstrated high pathological complete response rates (68%) and no increase in toxicity [47]. Subsequently, an open-label phase III trial was initiated, which demonstrated a significantly longer, time to loco-regional failure after R0-resection (HR 0.53; 95% CI 0.31–0.90; *p* = 0.017) [48]. Median OS was 5.1 years (95% CI, 3.7 to not reached) for cetuximab versus 3.0 years (95% CI, 2.2–4.2) for the control which was surgery alone (HR 0.73; 95% CI 0.52–1.01; *p* = 0.055) [48]. These findings are encouraging and in the absence of reported significant adverse effects, imply that the addition of cetuximab may be a useful adjunct in neoadjuvant therapy.

**Figure 2:**
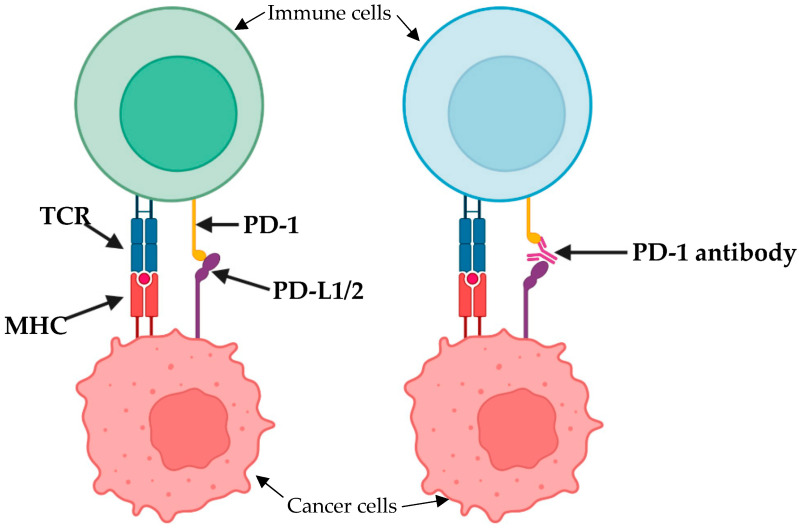
Binding of PD-1 to PD-L1 or PD-L2 downregulates the host immune response. Therefore, PD-1 or its associated ligands can be a potential target for monoclonal antibodies.

The programmed death factor receptor-1 (PD-1) is a regulatory immune checkpoint discovered in 1992 that is expressed in T, B, and NK cells and consists of two ligands, PD-L1 and PD-L2, which combine to downregulate a local immune response and can therefore promote carcinogenesis (Figure 2). Toripalimab is a recombinant humanised monoclonal antibody against PD-1, which acts to inhibit the binding of its ligands and therefore prevents a downregulatory response [49]. In 2021 a phase II trial involving toripalimab in conjunction with FLOT as a neoadjuvant regimen demonstrated efficacious outcomes with all 25% of patients demonstrating pCR and 42.9% demonstrating near pCR following surgery [50]. Another phase II trial was published in 2023, involving pembrolizumab (another PD-1 inhibitor) in conjunction with an mFOLFOX (5-fluorouracil, leucovorin, oxaliplatin) regimen [51]. Their results demonstrated that nearly 21% of patients achieved pCR with no unexplained toxicities. Although the use of PD-1 inhibitors is better studied in squamous cell carcinoma, these results are encouraging and suggest that PD-1 inhibitors may be another potential therapeutic option in adenocarcinoma.

**Figure 3:**
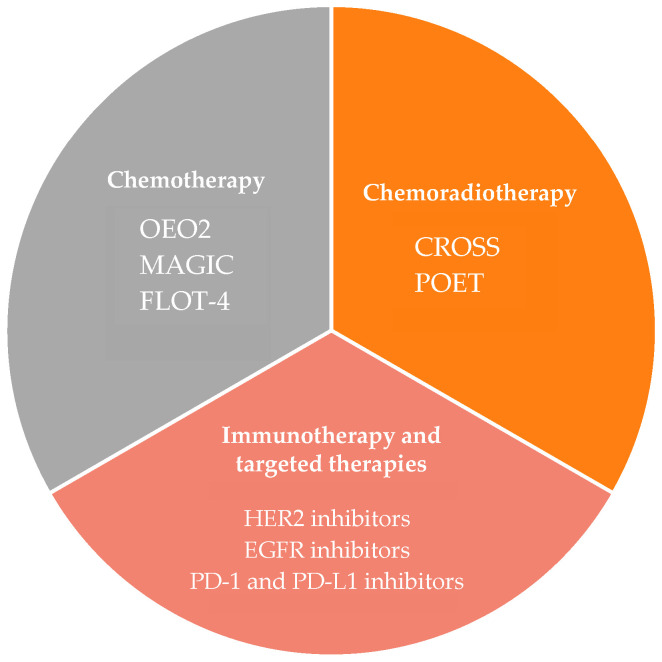
Summary of different Neoadjuvant strategies and key landmark trials to date [10,11,12,18,26,32,33,44,45,47,49,50,51].

The synergistic effects between immunotherapy, chemotherapy, and radiation have been reported in the literature. Chemotherapy can lead to immunosuppression through its effects on rapidly diving cells in the immune system; however, in noncytotoxic doses, it is hypothesised that immune activation can occur, which is likely as a result of changes in the composition of the tumour microenvironment through inhibiting the expression of certain immunosuppressor genes and expression of immunogenic antigens. An example is the case of increased HSP90 found in myeloma cells with the use of the proteasome inhibitor bortezomib [52]. It is thought that radiation-induced cell damage can further expose tumour antigens, making them visible to immune surveillance and promoting the priming and activation of cytotoxic T-cells. The addition of immunotherapy may further increase the sensitivity of tumours to radiation. With regard to oesophageal adenocarcinoma, encouraging results have been found with the addition of inhibitors of PD-1 and its associated ligands. The NCT03044613 trial evaluated the safety and efficacy of an induction regimen of nivolumab, followed by nivolumab administered alongside nCRT [53]. Data suggested acceptable toxicities without the delay of surgery and a high pCR of 40%. More recently, the results of the PERFECT trial, a phase II trial of atezolizumab (a PD-L1 inhibitor), in combination with neoadjuvant chemoradiotherapy have also been reported by ASCO in 2021 [54]. The chemoradiotherapy regimen used was identical to the CROSS trial and a total of 40 patients were enrolled in this study, of which 34 completed all cycles of atezolizumab therapy. The results revealed that nearly 30% of patients achieved PCR, higher than 23% in the CROSS study. However, is it of note that 6 patients (15%) experienced immune-related adverse effects; however, these were manageable and only occurred around the time of administration. Further trials are underway to research the effect of adding immunotherapy to a neoadjuvant regimen. Figure 3 details different neoadjuvant strategies and key trials to date.

## 6. Conclusions

This review has highlighted the different forms of neoadjuvant therapy that have been used for oesophageal and gastro-oesophageal junction adenocarcinomas in recent years. The most efficacious regimen still remains contentious in clinical practice and further high-powered randomised control trials are necessary to establish the optimal chemotherapy and radiation regimen, as well as the potential for immunotherapy to augment the neoadjuvant response in this patient cohort.
